# Combined proteomics, metabolomics and physiological analyses of rice growth and grain yield with heavy nitrogen application before and after drought

**DOI:** 10.1186/s12870-020-02772-y

**Published:** 2020-12-10

**Authors:** Jie Du, Tianhua Shen, Qiangqiang Xiong, Changlan Zhu, Xiaosong Peng, Xiaopeng He, Junru Fu, Linjuan Ouyang, Jianmin Bian, Lifang Hu, Xiaotang Sun, Dahu Zhou, Haohua He, Lei Zhong, Xiaorong Chen

**Affiliations:** Key Laboratory of Crop Physiology, Ecology and Genetic Breeding, Ministry of Education, College of Agronomy, Jiangxi Agricultural University, Jiangxi, 330045 China

**Keywords:** Rice, Nitrogen, Drought, Proteome, Metabolome

## Abstract

**Background:**

Nitrogen application can effectively mitigate the damage to crop growth and yield caused by drought. However, the efficiency of heavy nitrogen application before drought (NBD) and heavy nitrogen application after drought (NAD) to regulate rice response to drought stress remains controversial. In this study, we profiled physiology, proteomics and metabolomics in rice variety Wufengyou 286 of two nitrogen management modes (NBD and NAD) to investigate their yield formation and the mechanism of nitrogen regulation for drought resistance.

**Results:**

Results revealed that the yield of NBD and NAD decreased significantly when it was subjected to drought stress at the stage of young panicle differentiation, while the yield of NBD was 33.85 and 36.33% higher than that of NAD in 2017 and 2018, reaching significant levels. Under drought conditions, NBD increased chlorophyll content and net photosynthetic rate in leaves, significantly improved the activities of antioxidant enzymes such as superoxide dismutase (SOD), peroxidase and catalase, and decreased malondialdehyde (MDA) content compared with NAD. NBD promoted nitrogen assimilation in leaves, which was characterized by increased activities of nitrate reductase (NR) and glutamine synthetase (GS). In addition, NBD significantly increased the contents of osmotic regulatory substances such as soluble sugar, soluble protein and free proline. Gene ontology and KEGG enrichment analysis of 234 differentially expressed proteins and 518 differential metabolites showed that different nitrogen management induced strong changes in photosynthesis pathway, energy metabolism pathway, nitrogen metabolism and oxidation-reduction pathways.

**Conclusion:**

Different nitrogen management methods have significant differences in drought resistance of rice. These results suggest that heavy nitrogen application before drought may be an important pathway to improve the yield and stress resistance of rice, and provide a new ecological perspective on nitrogen regulation in rice.

**Supplementary Information:**

The online version contains supplementary material available at 10.1186/s12870-020-02772-y.

## Background

Drought is the main environmental factor leading to crop yield reduction, and the loss caused by drought is greater than the sum of other environmental stresses [1]. Different degrees of drought stress often lead to the closure of stomata in leaves, preventing the transfer of CO_2_ from the air to the intercellular and mesophyll cells of leaves [2, 3]. Ribulose-1,5-diphosphate (RuBP) is combined with low concentration of CO_2_, it is reduced by NADPH to form glyceraldehyde-3-phosphate (PGAld) after a series of reactions under the action of ribulose-1,5-diphosphate carboxylase (Rubisco). Since the assimilation of CO_2_ consumes NADPH and reduces the level of NADP^+^ electron transfer during the photosystemI (PSI), which restricts the leaf photosynthetic rate. Oxygen as an important electron acceptor in leaves leads to the rapid accumulation of reactive oxygen species (ROS) such as singlet oxygen (O_2_^−^), superoxide (^•^O_2_), hydroxyl (OH^− 1^) and hydrogen peroxide (H_2_O_2_) under adverse conditions, which has negative effects on antioxidant metabolism, resulting in cell peroxidation damage [4–6]. Antioxidant enzymes such as superoxide dismutase (SOD), peroxidase, catalase may play a central role in plant antioxidant metabolism under drought stress by regulating its gene expression and activity [7–9]. Drought stress also affects the activity of enzymes related to carbon and nitrogen assimilation, including sucrose synthase (SS), nitrate reductase (NR), glutamine synthetase (GS), glutamate dehydrogenase (GDH) and aminotransferase, causing changes in carbon and nitrogen compounds in plants and affecting the resistance of plants to drought stress [10–12]. Drought stress also promotes the expression of drought resistance genes in plant cells. It leads to the accumulation of functional proteins, such as membrane proteins (aquaporins), osmotic regulatory molecules (including sucrose, proline and betaine), macromolecular protective factors (such as heat shock proteins, molecular chaperones, mRNA binding proteins) [13, 14], and overproduction of regulatory proteins (including transcription factors, protein kinases) [15, 16].

In recent years, the role of N as a signaling factor in the regulation of stress tolerance in plants has attracted wide attention of researchers [17]. The function of N in regulating plant resistance to stress is closely related to the processes of N uptake and distribution [18]. The negative effects of drought stress on rice photosynthesis could be significantly improved by reasonably increasing the level of N application [19, 20]. Appropriately high nitrogen levels can increase the threshold of stomatal response to drought stress, maintain the integrity of photosynthetic [21], improve elasticity in root system development, and enhance plant antioxidant capacity [22]. Related studies have been reported in crops, such as rice [23], *Zea mays* [24], *Brassica campestris* [25] and *Triticum aestivum* [26], which showed that appropriately high level of nitrogen fertilizer can effectively alleviate drought stress and reduce yield loss. There are two main ways for farmers to apply nitrogen fertilizer to improve the drought resistance of crops. One is heavy application of nitrogen fertilizer before drought [27], which is often used to deal with seasonal drought. The other is heavy application of nitrogen fertilizer after drought [28], which is more common on “rain-fed fields” rice, mainly for remedial measures for irregular drought [29].

Proteomics and metabolomics are powerful tools for analyzing plant reactions to various environmental stimuli [30]. These methods can effectively improve the in-depth analysis of metabolic networks and protein regulation mechanisms [31]. Proteomics and metabolomics have been widely used to study the drought response pathways in the leaves of rice (*Oryza sativa* L.), maize (*Zea mays*) and durum (*Triticum durum*) wheat [32–34]. However, the efficiency of two methods of nitrogen application to regulate rice response to drought stress remains controversial, and there are few studies on the mechanisms of heavy nitrogen application before and after drought mitigation rice yield loss by combining proteomics, metabolomics and related physiological index. In this study, the rice variety Wufengyou 286 was used as the experimental material, and adopted two nitrogen management modes: heavy nitrogen application before drought (NBD) and heavy nitrogen application after drought (NAD). The young panicle differentiation stage was selected as the time point for drought treatment. To determine the efficiency of NBD and NAD in alleviating drought on the reduction of rice yield, the physiological index, proteomics, metabolomics analyses and yield were studied, which provide a theoretical reference for the management of nitrogen regulation of rice grown under drought conditions.

## Results

### Analysis of yield and yield components

In 2017, the yield of NBD per plant was significantly higher than that of NAD under drought stress, and they were significantly lower than CK (*p* < 0.05) (Table 1). The results showed that the young panicle differentiation stage was the sensitive stage to water deficit in double cropping early rice. Compared with NAD, the yield per plant of NBD was 33.85% higher, reaching significant levels (*p* < 0.05). From the perspective of yield components, compared with NAD, the seed setting rate of NBD was 16.09% higher (*p* < 0.05), the effective panicle number per plant, total grain number per panicle and 1000-grain weight of NBD was 6.03, 7.65 and 1.07% higher, respectively. The yield and yield components of different treatments in 2018 were basically similar to those in 2017 (Table 1). The yield per plant of CK was significantly higher than that of NBD and NAD (*p* < 0.05), and the yield per plant of NBD was 36.33% higher than that of NAD (*p* < 0.05). In terms of yield components, the total grains per panicle and seed setting rate of NBD were 11.73 and 14.61% higher than those of NAD (*p* < 0.05), respectively. Therefore, the yield and yield components for the NBD and NAD groups were comparable in the two evaluated years. The results showed that NBD was beneficial to reduce the yield loss of rice under drought stress, and the main reason for higher yield of NBD was the increase of seed setting rate and the number of grains per panicle.

**Table 1:** Yield and yield components of NAD and NBD in 2017 and 2018

Years	Treatment	Panicle length (cm)	Effective panicle	Total grains per panicle	Seed setting rate (%)	1000-grain weight (g)	Yield per plant (g)
2017	CK	18.91a	8.92a	131.42a	77.84a	20.47a	18.67a
	NBD	18.37a	8.44ab	127.84ab	66.65b	20.13a	14.47b
	NAD	18.03a	7.96b	118.75b	57.41c	19.93a	10.81c
2018	CK	18.66a	8.51a	133.75a	80.31a	20.28a	18.53a
	NBD	18.97a	7.78ab	130.93a	71.08b	20.32a	14.71b
	NAD	18.56a	7.41b	117.18b	62.02c	20.04a	10.79c

Note: Numerical values in the same column represent averages, and the different lowercase letters indicate statistically significant differences (*p* < 0.05), *n* = 7

### Analysis of dry matter, N uptake and distribution

The effects of different N fertilizer management on dry matter accumulation in rice stems, leaves and panicles were different under drought stress (Table 2). At the heading stage, the dry matter accumulation of stem, leaf and panicle of NBD was 21.15, 52.38 and 20.93% higher than that of NAD, respectively. The dry matter distribution of NAD to panicle was significantly lower than that of CK and NBD (*p* < 0.05). At mature stage, the dry matter accumulation in panicle of NAD decreased significantly, which was 27.46 and 41.77% lower than that of NBD and CK, respectively. The stem and leaf dry matter accumulation of NAD were all higher than that of CK and NBD to varying degrees. The percentages of the export percentage of stem and sheath matter (EPMSS) and transformed percentage of stem and sheath matter (TPMSS) were positive in the CK, while those were negative in NBD and NAD. The results showed that part of the stable chemical energy accumulated by photosynthesis in NAD was accumulated in the stem and was not effectively transported to the panicle.

**Table 2:** Dry matter accumulation and transport of ‘Wufengyou 286’ under heavy nitrogen application before and after drought in different periods. *n* = 5

Treatment	Heading stage (g)			Mature stage (g)			EPMSS (%)	TPMSS (%)
	Stem	Leaf	Panicle	Stem	Leaf	Panicle		
CK	9.06a	5.13a	3.90a	8.57c	3.67ab	22.72a	5.41	5.72
NBD	7.56b	4.48b	2.08b	9.11b	3.29b	17.66b	−20.50	− 17.01
NAD	6.24c	2.94c	1.72c	10.86a	3.97a	12.81c	− 74.04	−42.54

At heading stage, the nitrogen content of stem, leaf and panicle of CK and NBD was significantly higher than that of NAD (Table 3). Nitrogen accumulation in panicles, stems, leaves and above-ground parts of NAD was 56.34, 48.96, 46.21 and 48.81% lower than CK, and was 40.16, 31.77, 37.78 and 36.21% lower than NBD, respectively, and the difference were all significant (*p* < 0.05). At mature stage, the N accumulation in the panicles, stems and leaves of NAD plants was 11.57, 44.13 and 88.14% higher than that in NBD, respectively. The nitrogen accumulation ratio of the stem and leaf in NAD plants was significantly higher than that of CK and NBD (*p* < 0.05). Nitrogen accumulation in both panicle and above-ground parts of CK was significantly higher than that of NBD and NAD.

**Table 3:** N accumulation and accumulation ratio of ‘Wufengyou 286’ under heavy nitrogen application before and after drought in different periods

Growth stage	Treatment	N contents (%)			N accumulation (mg)			Above-ground N accumulation (mg)
		Panicle	Stem	Leaf	Panicle	Stem	Leaf	
Heading stage	CK	0.16a	0.34a	0.50a	55.64a	118.23a	173.55a	347.75a
	NBD	0.14b	0.32a	0.54a	40.91b	88.45b	149.78b	279.09b
	NAD	0.14b	0.34a	0.52a	24.48c	60.35c	93.20c	178.03c
Maturity stage	CK	0.73a	0.15b	0.12b	365.22a	74.69ab	58.55b	498.38a
	NBD	0.67ab	0.18ab	0.15b	226.25b	62.87b	51.37b	340.49b
	NAD	0.57b	0.21a	0.22a	252.44ab	90.62a	96.65a	439.71ab

### Analysis of photosynthesis-related parameters

**Fig. 1:**
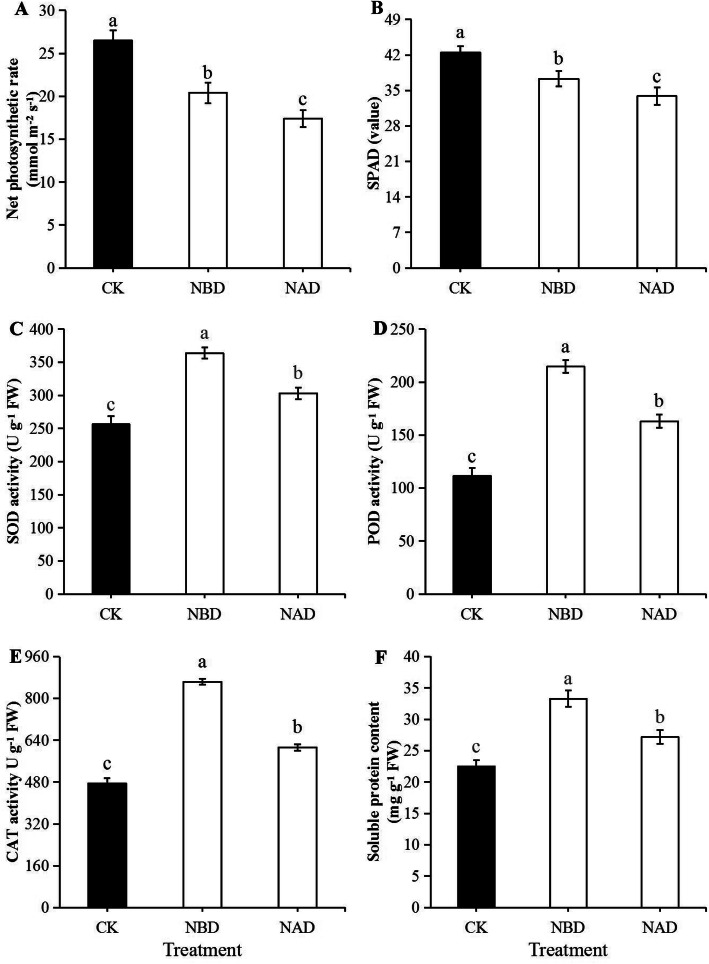
Net photosynthetic rate, SPAD value, and antioxidant enzyme activity and soluble protein content of ‘Wufengyou 286’ under heavy nitrogen application before and after drought. **a** Pn, **b** SPAD value, **c** SOD activity, **d** POD activity, **e** CAT activity and **f** Soluble protein content. Values are averages ± SD (n = 3), and statistically significant differences are indicated by lowercase letters (*p* < 0.05)

The net photosynthetic rate of NBD was 17. 29% higher than that of NAD, and reached a significant difference (Fig. 1a). The net photosynthetic rate of NBD and NAD was 22.98 and 34.33% lower than that of CK (*p* < 0.05), respectively. The stomatal conductance of CK, NBD and NAD were 436, 359 and 293 mmol/m^2^/s at the end of drought stress, and the differences between CK and both NBD and NAD were significant. The stomatal conductance of NBD was 21.52% higher than that of NAD (*p* < 0.05). The chlorophyll content of NBD was 10.03% higher than that of NAD, and the difference was significant (Fig. 1b). The chlorophyll content of CK was 13.94 and 25.96% higher than that of NBD and NAD (*p* < 0.05), respectively.

### Analysis of antioxidant enzyme activity and soluble protein

The SOD, peroxidase, and catalase activities of NBD were significantly increased compared to CK and NAD (Fig. 1c, d and e). The SOD activity of NBD was 20.09 and 40.92% higher than that of NAD and CK, respectively, showing significant difference. For peroxidase activity, NAD and CK were 24.04 and 48.11% lower than NBD, and the difference was significant. The catalase activity of NAD and CK was significantly lower than that of NBD by 29.08 and 44.95% (*p* < 0.05), respectively. In terms of soluble protein content, NBD was 22.52 and 42.66% higher than NAD and CK respectively, and there was significant difference (Fig. 1f).

### Analysis of osmotic adjustment substances

**Fig. 2:**
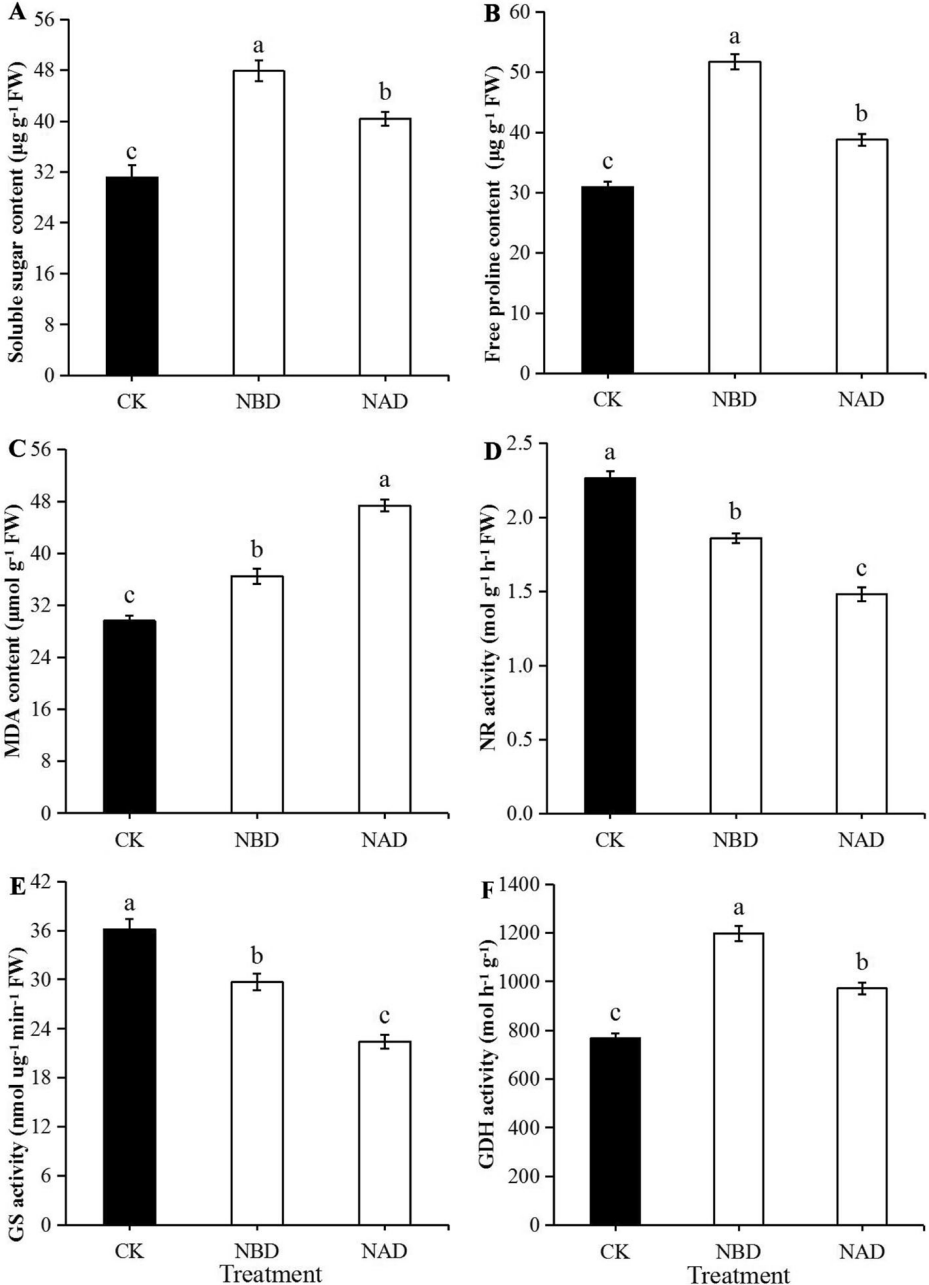
Soluble sugars, free proline and MDA content, and several nitrogen metabolism enzymes activities of ‘Wufengyou 286’ under heavy nitrogen application before and after drought. **a** Soluble sugar content, **b** Free proline content, **c** MDA content, **d** NR activity, **e** GS activity and **f** GDH activity. Values are averages ± SD (*n* = 3), and statistically significant differences are indicated by lowercase letters (*p* < 0.05)

After the end of the drought, the contents of soluble sugar and free proline in NBD were significantly higher than those in NAD and CK (Fig. 2a, b). The soluble sugar and free proline content of NBD were 18.38 and 33.33% higher than that of NAD, and the difference was significant (*p* < 0.05). NBD and CK had significantly lower MDA content than NAD (Fig. 2c), with NBD being 22.87% lower than NAD.

### Analysis of enzymatic activity related to N metabolism

The NR and GS activities of NBD and CK were significantly higher than those of NAD (Fig. 2d, e). In terms of enzyme activity, the NR activity of NBD and NAD was 17.88 and 34.58% lower than that of CK, and the activity of GS was 17.92 and 38.01% lower than that of CK, respectively. The NR and GS activities of NBD were 25.53 and 32.31% higher than those of NAD, respectively, showing significant differences (*p* < 0.05). The GDH activity of NAD and CK was significantly lower than that of NBD (Fig. 2f), with NAD having 18.85% lower GDH activity than NBD.

### Proteomic identification analysis with TMT

**Fig. 3:**
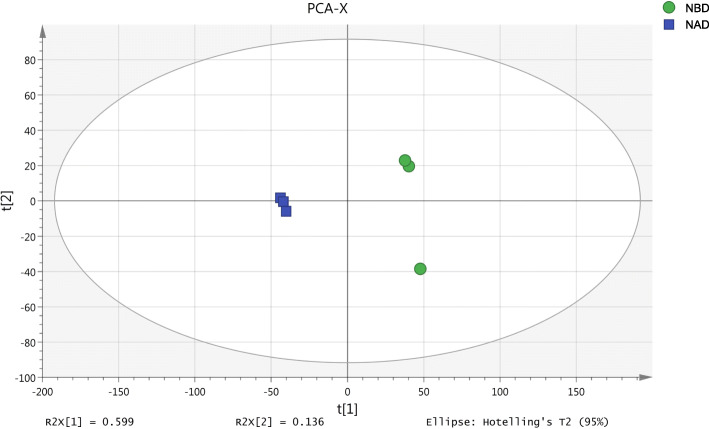
Principal component analysis of ‘Wufengyou 286’ under heavy nitrogen application before and after drought using individual replicates

**Fig. 4:**
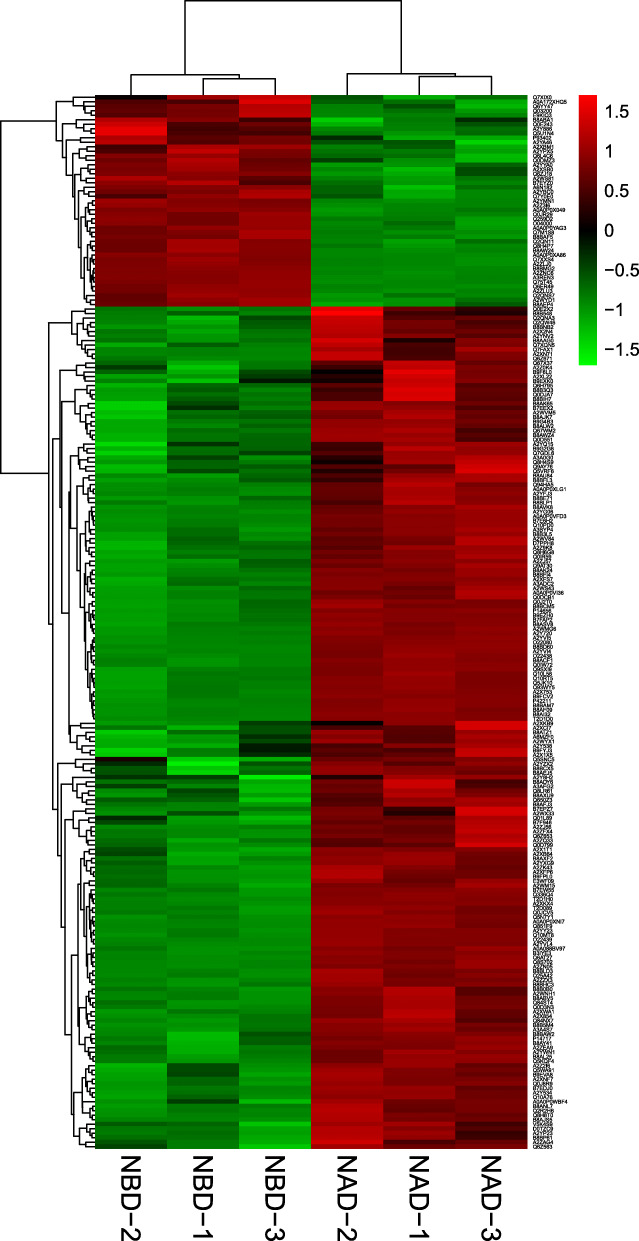
Hierarchical clustering of proteomic changes in ‘Wufengyou 286’ under heavy nitrogen application before and after drought. The columns correspond to repeats of the NAD and NBD groups and the rows correspond to differentially expressed proteins. Red and green represent high and low expression of DEPs for NBD and NAD respectively

This proteomic experiments were conducted by TMT labeling of rice leaves subjected to the two treatments of NBD and NAD. After each sample was detected by LC-MS/MS, the quantity of qualitative protein and quantitative protein were 4254 and 3892 respectively. The samples labeled by enzymolysis of each treatment were analyzed via LC-MS and searched in database. A total of 3548 trusted proteins were screened by TMT experiment. Based on the comparative analysis of NBD and NAD samples, it was found that PCA score could explain 73.5% of the separation between NAD and NBD (Fig. 3), so it was credible for PCA to explain the protein differences between NAD and NBD samples. In addition, we visualized the differentially expressed proteins (DEPs) of different points in NBD and NAD through cluster analysis (Fig. 4), and the results further suggested that the data were trusted.

### Functional analysis of DEPs

**Fig. 5:**
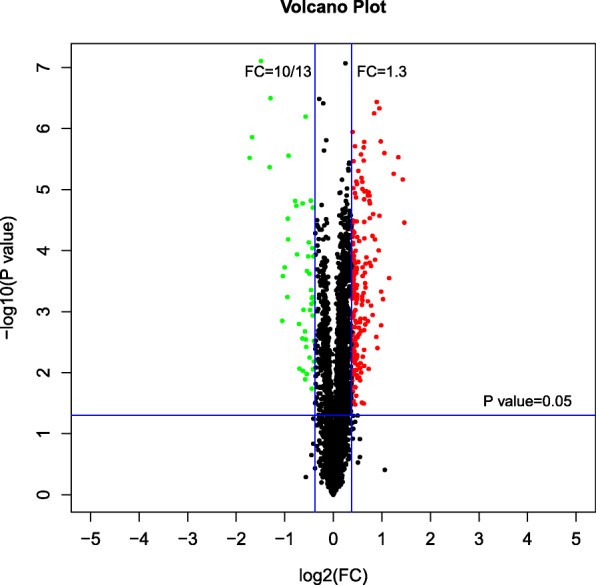
Volcano plot of proteomic changes in ‘Wufengyou 286’ under heavy nitrogen application before and after drought. Green dots represent DEPs that are down-regulated in NBD and NAD, red dots represent DEPs that are up-regulated, and black dots represent DEPs that are not significantly different

Based on the selected trusted protein, proteins with fold change > 1.3 were regarded as up-regulated (*p* < 0.05), and those with fold change < 10/13 were considered down-regulated (*p* < 0.05) [35]. Consequently, the DEPs were considered as NBD and NAD responsive proteins, and 234 DEPs were derived between the NAD and NBD groups (47 up-regulated and 187 down-regulated) (Dataset S1). In DEPs between NAD and NBD, 36 up-regulated and 105 down-regulated proteins have been functionally annotated by gene ontology, while the rest of the DEPs had not been annotated. Figure 5 showed the volcano plot of the DEPs of NAD-NBD.

**Fig. 6:**
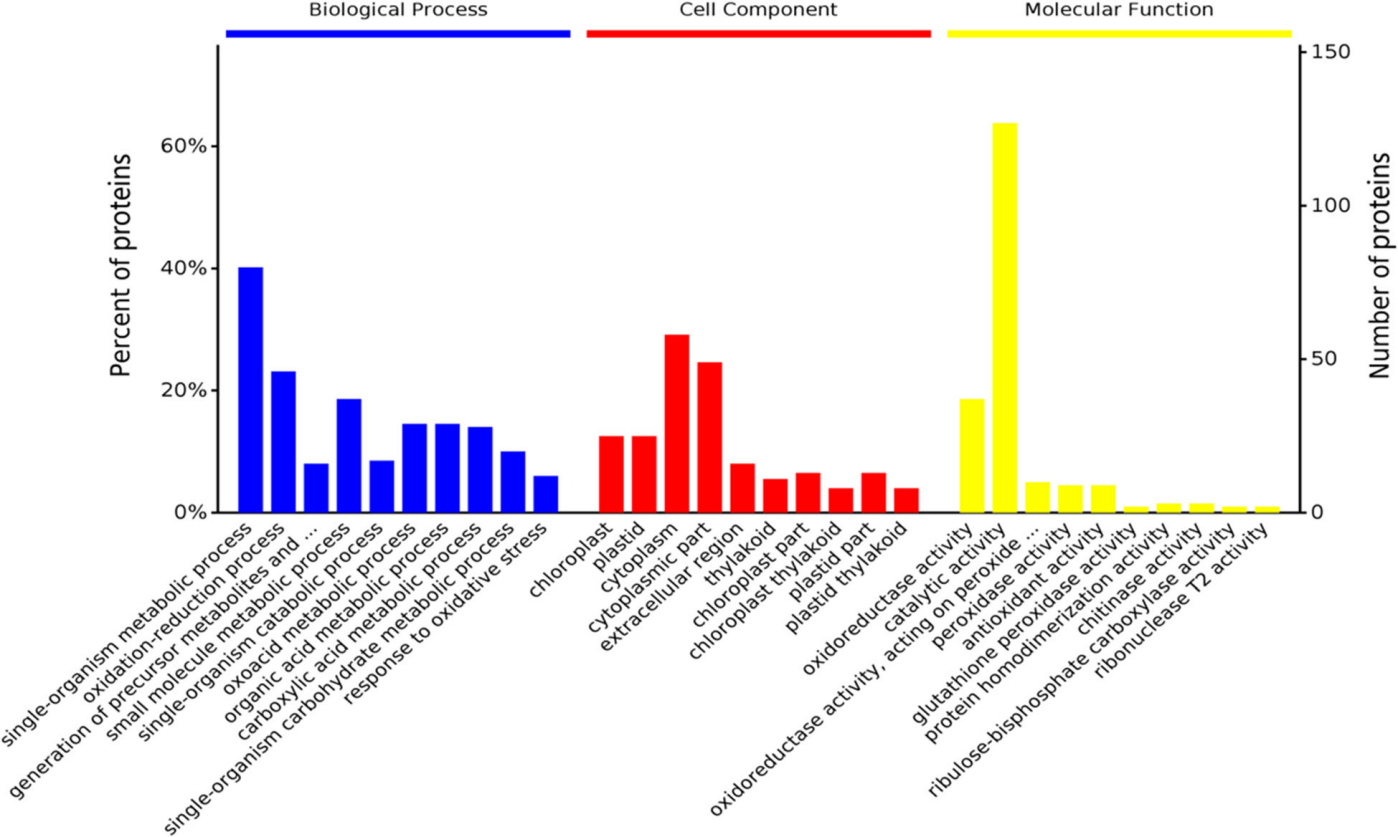
Gene ontology enrichment of ‘Wufengyou 286’ under heavy nitrogen application before and after drought. The horizontal coordinates are sorted from left to right based on the *p*-values of the entries in the different categories and the vertical coordinates represent the number of DEPs and their percentage of the total number of DEPs

In order to further analyze the functional characteristics of DEPs between NBD and NAD, the 141 DEPs were first annotated for gene ontology function and then analyzed at three levels: biological process, cellular component and molecular function. According to the significance analysis of the number of DEPs genes, the top three items in biological process were single-organism metabolic, oxidation-reduction, and small molecule metabolic process. The top three items in cellular component were cytoplasm, cytoplasmic part and chloroplast. The top three items in molecular function were catalytic activity, oxidoreductase activity and acting on peroxide as acceptor, peroxidase activity (Fig. 6). The KEGG pathway analysis was then performed on DEPs, and the results showed that the main KEGG pathways involved in NAD and NBD included: biosynthesis of secondary metabolites, carbon metabolism, biosynthesis of amino acids, glutathione metabolism, carbon fixation in photosynthetic organisms and nitrogen metabolism (Fig. S1). Furthermore, the protein interaction between differential proteins and KEGG pathways was analyzed, and the protein interaction network map was drawn (Fig. S1). The results showed that the partially up-regulated differential proteins A0A088BV97, O22438, and Q0D3N3 interacted with KEEG pathways including secondary metabolite biosynthesis, carbon metabolism, metabolic pathways, and amino acid biosynthesis in NAD and NBD.

### Metabolite profiles

**Fig. 7:**
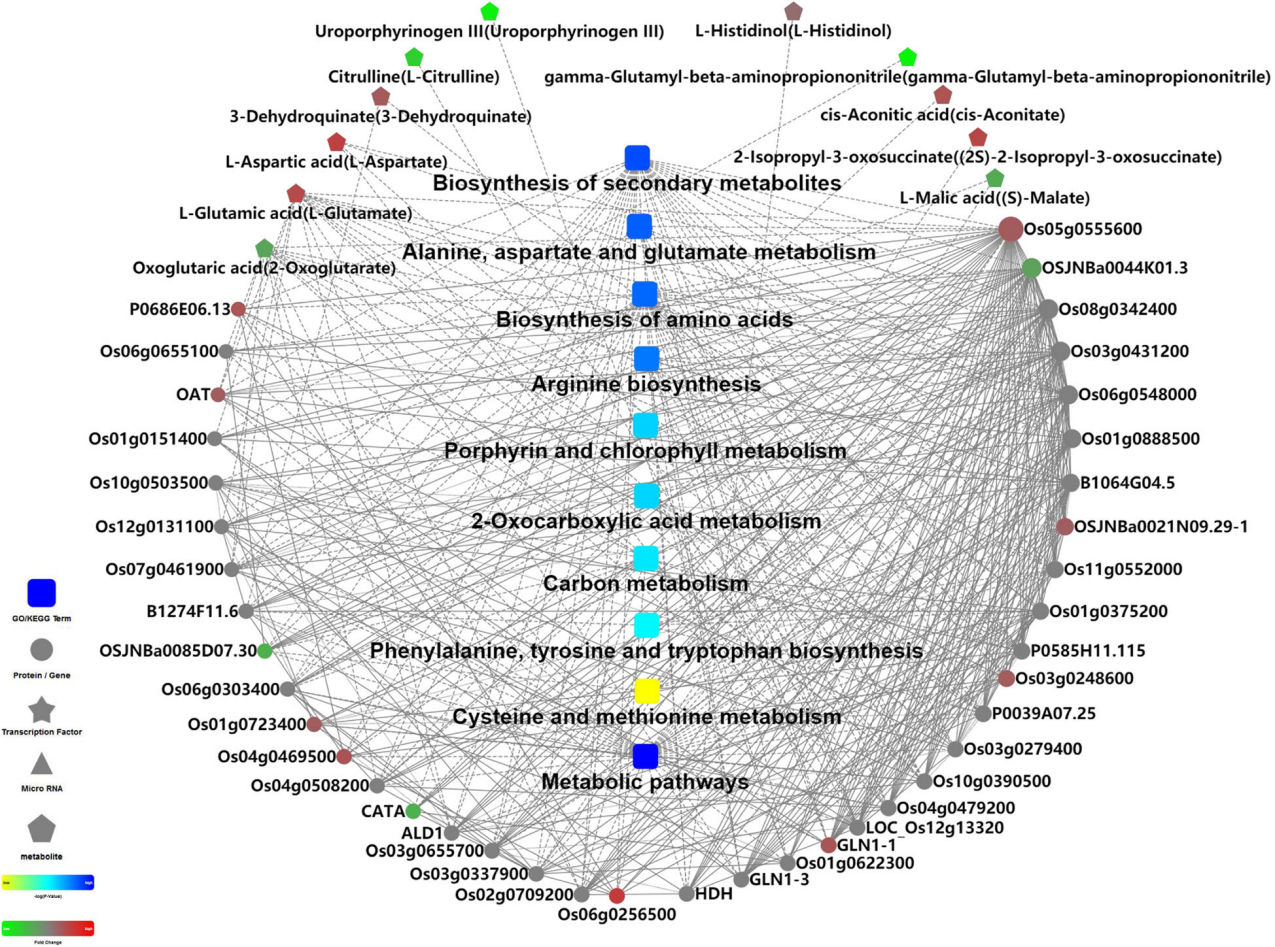
DEPs, DMs and related enzymes and metabolic pathway interactions analysis of ‘Wufengyou 286’ under heavy nitrogen application before and after drought

Firstly, PCA was performed on the NAD and NBD samples, and the results showed that the model was credible in explaining the metabolic differences between the two groups (Fig. S2A); the PLS-DA model could better explain the differences between the NAD and NBD samples (Fig. S2B); the NAD and NBD samples had spectral separation on the OPLS-DA score plot (Fig. S2C), and finally the OPLS-DA model was tested with 200 response ranking tests, which showed that this model was not overfitting (Fig. S2D). The screening criteria of differential metabolites (DMs) and differential proteins were same. Consequently, the DMs were considered as NBD and NAD responsive metabolites. In total, 518 DMs (296 up-regulated and 222 down-regulated) were screened from the rice leaves between the NAD and NBD groups (Dataset S2). Then, the pathway enrichment analysis was performed using the KEGG ID of the DMs, which showed that DMs were significantly enriched in porphyrin and chlorophyll metabolism, citrate cycle, metabolic pathways and carbon fixation in photosynthetic organisms pathway. More information about DMs was contained in Dataset S2. In order to more clearly visualize the differences in the expression patterns of metabolites in NAD and NBD samples, hierarchical clustering was performed using the expression of DMs for each group of samples (Fig. S3). This showed that the NAD and NBD plants had different responses to water stress at the metabolite level. The significant enrichment of the KEGG pathways in DEPs and DMs mapped to the KEGG database were shown in Table 4. Furthermore, the interaction network map of differential proteins, DMs and metabolic pathways was constructed by omicbean platform (Fig. 7), and the metabolic pathways were ranked according to their significance: metabolic pathways; biosynthesis of secondary metabolites; alanine, aspartate and glutamate metabolism; biosynthesis of amino acids; arginine biosynthesis; porphyrin and chlorophyll metabolism and carbon metabolism pathways.

**Table 4:** The KEGG pathways involving both DEPs and DMs of ‘Wufengyou 286’ under heavy nitrogen application before and after drought

Pathway Name	Pathway ID	Number of proteins		*p*-value
		Mapping	All	
Metabolic pathways	osa01100	132	1560	2.95E-19
Biosynthesis of secondary metabolites	osa01110	85	834	9.54E-15
Alanine, aspartate and glutamate metabolism	osa00250	18	43	1.20E-13
Biosynthesis of amino acids	osa01230	37	207	3.10E-13
Arginine biosynthesis	osa00220	14	28	3.56E-12
Porphyrin and chlorophyll metabolism	osa00860	11	37	5.39E-07
2-Oxocarboxylic acid metabolism	osa01210	12	46	7.79E-07
Carbon metabolism	osa01200	27	235	9.23E-06
Phenylalanine, tyrosine and tryptophan biosynthesis	osa00400	9	42	1.07E-04
Cysteine and methionine metabolism	osa00270	13	96	4.66E-04
Glyoxylate and dicarboxylate metabolism	osa00630	10	63	5.99E-04
Tropane, piperidine and pyridine alkaloid biosynthesis	osa00960	5	16	6.22E-04
Nitrogen metabolism	osa00910	6	28	1.58E-03
Glutathione metabolism	osa00480	10	73	1.94E-03
Carbon fixation in photosynthetic organisms	osa00710	10	73	1.94E-03
Citrate cycle (TCA cycle)	osa00020	8	51	2.29E-03
Nicotinate and nicotinamide metabolism	osa00760	4	15	4.29E-03
Glycine, serine and threonine metabolism	osa00260	8	57	4.67E-03
Pyrimidine metabolism	osa00240	12	110	5.00E-03
Phenylalanine metabolism	osa00360	6	35	5.16E-03
Flavone and flavonol biosynthesis	osa00944	2	3	6.39E-03
Tyrosine metabolism	osa00350	6	38	7.81E-03
Arachidonic acid metabolism	osa00590	3	10	9.60E-03

Note: The enrichment pathways are ranked in descending order of *p*-value

## Discussion

### Yield, and physiological and biochemical responses to heavy nitrogen application before and after drought

Drought stress usually results in the decrease of grain number per panicle, seed setting rate and yield of rice [20]. Previous studies have shown that different nitrogen application rates and nitrogen management can regulate crop water and yield under drought stress [19, 36]. Appropriately high levels of nitrogen under drought conditions promote root development and reduce yield loss in rice [37]. In this study, it was found that the yield of NBD was significantly higher than that of NAD in 2017 and 2018. The increased yield of NBD was mainly due to increased seed setting rate and number of grains per panicle (Table 1). Increasing the level of N fertilizer application can effectively enhance the antioxidant capacity of plants [38]. The results showed that the peroxidase, SOD and catalase activities of NBD and NAD were significantly higher than those of CK, while the SOD, peroxidase and catalase activities of NAD were significantly lower than those of NBD. A large amount of MDA accumulation after drought stress showed that the membrane structure of rice plants was damaged to a certain extent. The increase of antioxidant enzyme activity in NBD was beneficial to alleviate leaf injury, resulting in a significant decrease in MDA content compared with NAD (Fig. 2c). Appropriately high N levels can coordinate carbon and nitrogen metabolism and mitigate the inhibition of photosynthesis by water stress [21]. This study showed that the chlorophyll content and net photosynthetic rate of the NBD treatment were significantly higher than those of the NAD treatment. It also showed that NBD could ameliorate the adverse effects for drought stress on rice photosynthesis, and this mitigation effect was beneficial to the production and accumulation of dry matter, which was consistent with the yield and dry matter results of NBD (Table 2). Under drought stress, there is a significant difference in the distribution of dry matter among leaves, stems, panicles and roots in rice, which also alters the relationship between root-crown ratios [39]. At heading stage, the dry matter accumulation of stem, leaf and panicle of NBD was significantly higher than that of NAD. At the mature stage, the dry matter accumulation in panicle and TPMSS of NBD were higher than that of NAD, this provides a guarantee for the increase of yield by NBD. The application of high levels of nitrogen enhances the assimilation of NO_3_^−^ and NH_4_^+^ and increases the activity of enzymes related to nitrogen metabolism [22]. This study showed that the activities of NR and GS and the contents of soluble sugar and free proline in NBD treatment were significantly higher than those in NAD, and GDH activity significantly lower than those in NAD. This was consistent with that the nitrogen accumulation in each organ of NBD was significantly higher than that of NAD at heading stage (Table 3). The results showed that NBD was beneficial for increasing the activity of enzymes related to nitrogen metabolism and the content of osmotic adjustment substances, and plays an important role in rice adaptation to drought stress. Huang et al. found that the accumulation of amino acids and soluble proteins was beneficial to the improvement of plant drought resistance [40], and the results of our study were consistent with them. Therefore, NBD is advantageous for maintaining the photosynthetic potential of rice and coordinating the carbon and nitrogen metabolism of rice, which plays an important role in improving rice stress tolerance and yield formation. In summary, the injury mechanism and drought resistance mechanism of rice under water stress is a complex process. There are three main strategies for plants to deal with water shortage: drought escape, drought avoidance and drought tolerance [41], which will affect the development of plants response to drought stress. NBD promotes the accumulation of osmotic regulatory substances and increases antioxidant enzyme activity by regulating the mechanism of drought tolerance in rice, which provides a guarantee for the maintenance of physiological activities of plants. In addition, severe drought caused intense injury to rice at the panicle differentiation stage, which may result in irreversible damage to plants, and thus two modes of N application were unable to compensate for yield to normal levels. Future studies aim at different total nitrogen fertilizer levels to regulate rice yield and stress resistance pathways, which will better clarify the exact role of nitrogen fertilizer application in maintaining yield formation under water stress.

### Photosynthesis responses to heavy nitrogen application before and after drought

Drought affects plants primarily by affecting photosynthesis, and the responses of photosynthesis to drought stress are extremely complex [42]. Under mild and moderate drought stress, photosynthetic assimilation capacity is directly affected by reduced stomatal and mesophyll conductances leading to reduced chloroplast utilization of CO_2_, or as a result of changes in photosynthetic metabolism [43, 44]. Previous studies have found that the photosynthetic rate of plants increases with increasing N concentration, and decreases with decreasing soil moisture content [45]. The effect of water-N interaction on photosynthesis has been observed in many crops [46]. Light-harvesting chlorophyll a/b-binding proteins (LHCBs) are the apolipoprotein of the photosystem II (PSII) complex. These chloroplast/thylakoid proteins are encoded by nuclear genes, and their expression is mainly regulated by environmental and developmental factors [22]. In this study, the chlorophyll a-b-binding proteins (protein ID: A2WS81, A2YMN1, Q7M1S8) of NBD were significantly up-regulated compared to NAD (*p* < 0.05). In agroecosystems, crop photosynthetic capacity limitations can be mitigated by increased N fertilization, which can allocate more N to photosynthetic proteins to further improve crop photosynthetic nitrogen use efficiency [47]. This study showed that the content of light-regulating proteins (protein ID: Q03200, A2Y886) in NBD was substantially higher than that of NAD. This was consistent with the results of significantly higher N accumulation in NBD leaves than that of NAD at heading stage. Severe drought stress leads to non-stomatal limitation of photosynthesis through oxidative stress, inhibiting the function of rubisco enzymes and electron transfer complexes in leaves, thus affecting the photosynthetic mechanism [48]. In this study, the expression of ribulose bisphosphate carboxylase related protein (protein ID: A6N182) in NBD was significantly up-regulated (*p* < 0.05). The results showed that after rice suffered severe drought during young panicle differentiation, the parameters such as chlorophyll and net photosynthetic rate decreased may by limiting stomatal opening, inhibiting PS II photoreaction and electron transport process and reducing intercellular carbon dioxide concentration [49]. Heavy nitrogen application before drought greatly contributes to increase the synthesis and expression of photosynthetic pigments and relate proteins such as ribulose diphosphate carboxylase, increased stomatal opening, and maintained high electron transfer rate and rubisco enzyme activity, thereby protecting the biochemical processes of photosynthesis and enhancing the adaptability of rice to drought stress.

### General metabolism responses to heavy nitrogen application before and after drought

The regulation of N on plant drought tolerance is related to applied N levels and stress intensity [50]. Previous studies have found that appropriate high nitrogen fertilizer levels promote the absorption and distribution of nitrate in plant roots and effectively enhance plant resistance to stress [51]. This study showed that nitrogen accumulation in all organs and above ground parts of NBD was significantly higher than that of NAD at heading stage. Nagy et al. considered that GS can be used as an important metabolic index of plant drought resistance [52]. Overexpression of NR and GS1 genes can significantly maintain plant N assimilation ability and improve plant drought resistance ability [53]. In this study, it was shown that the NBD treatment significantly increased the expression of amino acid synthesis and nitrogen metabolism-related proteins (protein ID: Q2QN11, P93402) compared to NAD, which was consistent with the increased activities of key nitrogen metabolism enzymes such as NR and GS in NBD. This indicated that nitrogen metabolism enzymes in NBD promoted the assimilation of nitrate and ammonium and synthesized the osmotic substance proline and other compounds to maintain cellular water balance. In addition, the reduction of NO_3_^−^ in leaves can consume energy more efficiently, which plays an important role in enhancing the thermal energy dissipation of leaves under drought conditions and alleviating the inhibition of excess light energy on photosynthesis [54]. Under water stress, NH_4_^+^ accumulation in plant tissues results from increased protein hydrolysis and enhanced light respiration, and excess NH_4_^+^ accumulation is toxic to plants [55]. GDH can directly catalyze the condensation of NH_4_^+^ with α-ketoglutarate (α-OG) to form Glu in the NH_4_^+^ assimilation pathway, thus reducing the adverse effects of NH_4_^+^ on rice [56]. The present study showed that the GDH activity of NBD was significantly higher than that of NAD, and the expression of related metabolic enzyme proteins (protein ID: Q7Y0E0) was significantly increased. As an important element in the synthesis of organic osmotic regulation substances, nitrogen plays a key role in improving plant agronomic and physiological properties [50]. Zhong et al. demonstrated that higher nitrogen application levels enhanced rice adaptation to drought stress, as evidenced by increased osmoregulators and increased proline content in leaves [22]. This study showed that NBD significantly increased the expression of sucrose synthase associated protein (protein ID: A2YA46) and carboxylic acid reduction-associated protein (protein ID: A6N182) compared to NAD, consistent with an increase in soluble sugars and free proline content. This indicated that NBD promoted free proline as molecular chaperone to stabilize the integrity and protein structure of cell membrane, and increased osmotic regulating substances such as soluble sugar to regulate cell osmotic potential.

### Antioxidant responses to heavy nitrogen application before and after drought

**Fig. 8:**
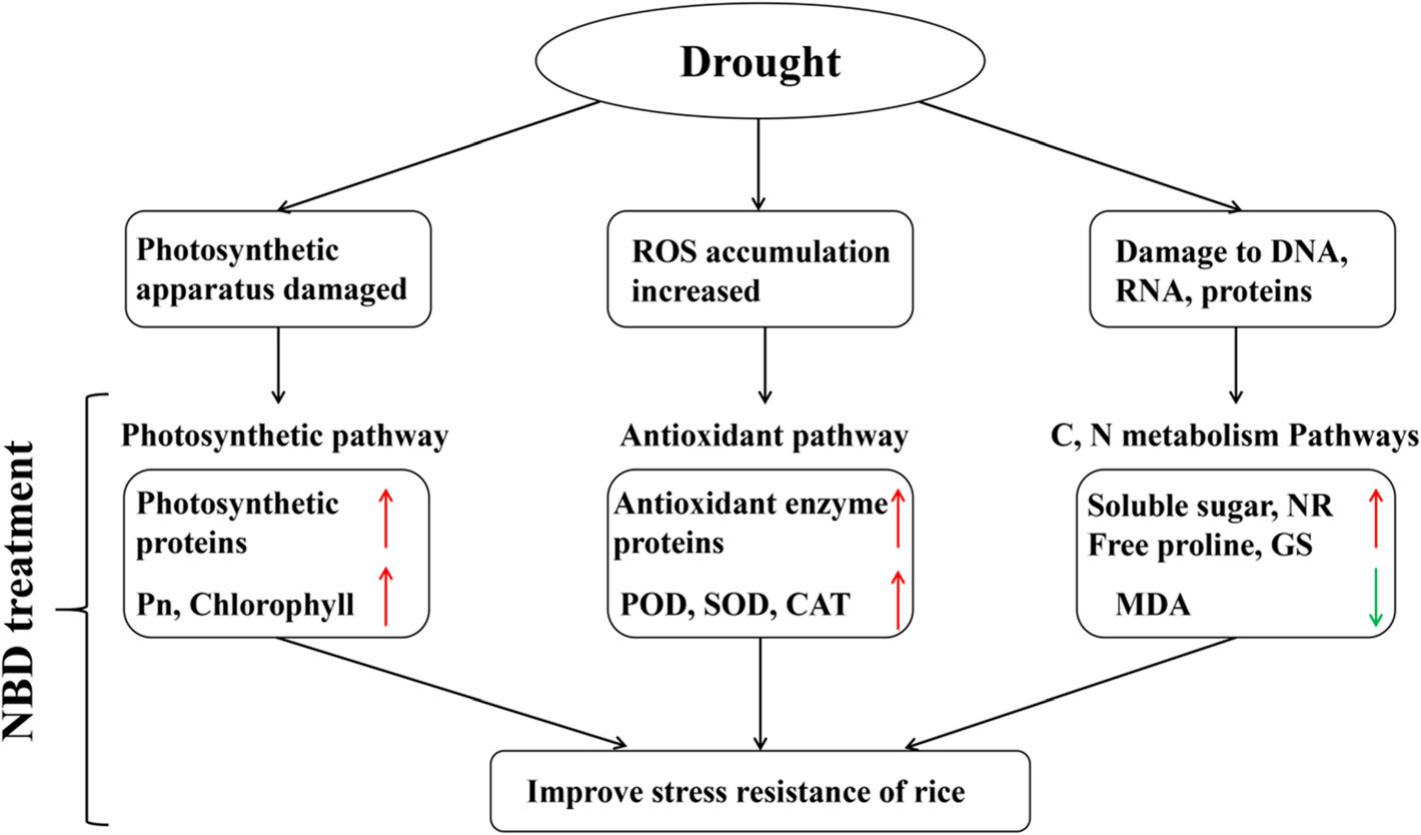
Response of ‘Wufengyou 286’ to heavy nitrogen application before drought stress. Red arrows indicate an increase in response or content compared to NAD, and green arrows indicate a decrease in response or content compared to NAD

The blockage of electron transfer in the respiratory chain under drought stress resulted in excessive accumulation of reactive oxygen species such as H_2_O_2_, O_2_**·**ˉ and nitrogen oxides in the cells [9], which induced severe oxidative stress leading to oxidative damage of membrane structures and organelles in the cells [4]. Drought stress increased the activities of catalase, SOD and glutathione peroxidase in the antioxidant enzyme system in plants [38]. Increasing the level of N fertilizer application could further enhance the antioxidant capacity of plants, inhibit membrane peroxidation and alleviate the aggravation of leaf peroxidative damage [57]. This study showed that the biological processes of the NBD and NAD were significantly enriched in response to redox processes, reactive oxygen metabolism processes, and oxidative stress, as evidenced by differential proteins gene ontology enrichment (Fig. 6). NBD treatment significantly increased the expression of peroxidase associated protein (protein ID: A2YPX5, A3REN3, Q5U1N4) compared with NAD, which was consistent with the increase in the activities of antioxidant enzymes such as peroxidase, SOD and catalase in NBD. MDA is the final product of membrane lipid peroxidation, and the change of its content is one of the important indicators of the degree of plasma membrane damage [6]. This study showed that NBD significantly reduced the expression of lipoxygenase related protein (protein ID:A2XL22), which was consistent with the reduction of MDA content in NBD. This indicated that severe drought had induced disintegration of leaf cell membranes and electrolyte leakage in the plants, NBD can contribute to mitigate the involvement of lipoxygenases in membrane lipid peroxidation and reduce damage to the cell membrane system. Glutathione system plays an important role in the scavenging of reactive oxygen species and biological protection [58]. This study showed that NBD significantly up-regulated the expression of glutathione peroxidase-related proteins (protein ID: Q10L56, B8ASV8) compared with NAD. Glutathione is an essential component of the ascorbic acid-glutathione cycle, and increased glutathione peroxidase activity in NBD promotes the maintenance of intracellular redox potential under severe drought, and also catalyzes the reduction of H_2_O_2_ and lipid peroxidation, thereby protecting leaves from reactive oxygen species toxicity produced by environmental stress. This indicated that multiple mechanisms were involved in the regulation of N on the antioxidant system of plants under drought conditions (Fig. 8). Heavy nitrogen application before drought enhanced the activities of peroxidase and SOD in antioxidant enzyme system, increased the content of reduced glutathione and ascorbic acid/dehydroascorbic acid ratio in antioxidant non-enzyme system. This plays an essential role in maintaining cell redox state and signal transduction under drought stress, and effectively alleviates oxidative stress to some extent, and finally protected the biochemical effect of photosynthesis and increased yield.

## Conclusions

Different nitrogen management methods have significant differences in drought resistance of rice. The results showed that heavy nitrogen application before and after drought had different effects on yield, physiological and biochemical parameters, protein and metabolic mechanisms under drought stress at young panicle differentiation stage of rice. Compared to heavy nitrogen application after drought at the stage of young panicle differentiation for rice variety Wufengyou 286, heavy nitrogen application before drought significantly increased the yield, net photosynthetic rate, chlorophyll and osmoregulatory substance content of leaves, and the activities of antioxidant enzymes and nitrogen metabolic enzymes were also increased in varying degrees, which helped to alleviate the inhibition of photosynthesis and leaf lipid peroxidation, and ultimately improved the yield. 234 DEPs and 518 DMs were closely associated with photosynthetic pathways, redox pathways, sucrose metabolism and nitrogen metabolism pathways, as well as resistance associated proteins. These results suggest that heavy nitrogen application before drought may be an important pathway to improve rice drought tolerance, and provide a new ecological perspective on the impact of nitrogen on the regulation of rice water.

## Methods

### Plant materials

Wufengyou 286 (Wufeng A/Zhonghui 286, *Oryza sativa* L.) is one of the rice varieties with large planting area in Jiangxi Province, the main producing area of double-season rice in South China. In this study, the experimental material Wufengyou 286 was bred and provided by China Rice Research Institute. In 2017 and 2018, the experiment was performed at Jiangxi Agricultural University in Jiangxi Province, China (28°45′N, 115°49′E, average annual rainfall: 1650 mm, and annual average temperature: 17.7 °C). Rice plants were cultivated in plastic buckets with 25.0 cm high, 30.0 cm upper diameter and 23.5 cm bottom diameter. The experimental soil was 0 ~ 20 cm topsoil, which came from an unused wasteland for several years. The physical and chemical characteristics of the soils in 2017 and 2018 were shown in Table 5. After the soil was dried naturally, it was crushed with a FT-1000A soil disintegrator (Shandong Able Instrument Co., Ltd. China) and then screened with a 100 mm mesh. The soil was presubmerged for 2 weeks before transplanting. In the four-leaf period, well-grown and consistent rice seedlings were transplanted into buckets, and planted according to the specifications of three seedlings per bucket and one seedling per hole. Combined with the nutrition requirement of plants, 5.0 g calcium-magnesium-phosphate fertilizer and 1.0 g potassium-chloride were applied in every bucket as basal fertilizers. Using urea as nitrogen source, 5.0 g per bucket was applied and was equal to 180 kg·hm^− 2^. In accordance with the experimental design, mixed seedling stage fertilizer was added to the buckets before transplanting, and the rest of the nitrogen fertilizer was applied in combination with irrigation at each growth stage. Before transplanting, the field was managed uniformly, and after transplanting, the water, diseases and insect pests were managed according to the mode of high yield cultivation. Pay attention to the weather changes after each fertilization, the plastic buckets were moved to a rainproof shed in the case of heavy rain to avoid the loss of fertilizer elements caused by water overflow, and the buckets were moved directly back to outside space after the rain stopped.

**Table 5:** Physical and chemical properties of the soil in 2017 and 2018

Years	Soil pH	Organic matter (g kg^−1^)	Total nitrogen (g kg^−1^)	Available nutrient (mg kg^−1^)		
				N (nitrogen)	P (phosphorus)	K (potassium)
2017	5.9	21.44	1.24	110	12.7	85.2
2018	5.7	23.18	1.12	101	10.8	90.8

### Experimental treatments

According to the results of previous studies, double-cropping rice is extremely vulnerable to drought stress at the young panicle differentiation stage [59]. Therefore, the Wufengyou 286 variety was selected for the drought stress at the stage of young panicle differentiation. In 2017, the early rice was sown on March 16 and transplanted on April 26. Two treatments applied basal fertilizer on April 25, tiller fertilizer on May 3, and panicle fertilizer on June 14. In 2018, the early rice was sown on March 15 and transplanted on April 25. Basal fertilizer was applied on April 24, tiller fertilizer on May 2, and panicle fertilizer on June 13, respectively. According to the experimental design, for NAD plants, 1 g nitrogen fertilizer was applied as base fertilizer, 1 g nitrogen fertilizer was applied as tiller fertilizer, and 3 g nitrogen fertilizer was applied as panicle fertilizer. For NBD and control treatment (CK), 1 g nitrogen fertilizer was applied as base fertilizer, 3 g nitrogen fertilizer was applied as tiller fertilizer, and 1 g nitrogen fertilizer was applied as panicle fertilizer (Table 6). The CK plants maintained 3 ~ 5 cm water layer during the growth period, and all treatment groups were water-broken one week before harvesting. The average temperature during drought stress in 2017 and 2018 was 26.1 °C and 24.9 °C, and the average relative humidity was 81.6 and 77.1%, respectively. For drought stress, the water contained in the buckets of NAD and NBD plants was poured out by manual tilt and then moved into the glass house for ten days of drought (the growth point of the young stem grew villi, and the length of the young panicle was 1 mm). Soil moisture content monitoring was based on a JK710 soil moisture meter (measurement range: 0–50%, Beijing Huaxi Techtron Technology Co., Ltd.). Progressive drought stress was applied to the soils of different treatment groups, and the soil moisture content of different treatment groups was measured every evening. As the drought stress progressed, the leaves of the different treatment groups began to wilt and curl, and the soil surface became clumpy and cracked. Drought stress was continued for two days after soil moisture content < 5% (m^3^/m^3^) reached the severe drought criterion for NAD and NBD groups (simulated severe drought). After the end of the drought, NAD plants and NBD plants were removed from the glass house for rewatering and application panicle fertilizer, respectively (Table 6). According to the randomized block design, both the NAD and NBD treatments were performed with 3 replicates, each containing 20 buckets.

**Table 6:** N supply timeline and drought stress for heavy nitrogen application before and after drought

Treatments	April 25	May 2	June 2 to June 12	June 13
CK	Applied 1 g N	Applied 3 g N	3 ~ 5-cm water layer	Sampled and applied 1 g N
NBD	Applied 1 g N	Applied 3 g N	Drought duration	Sampled and rewatering Applied 1 g N
NAD	Applied 1 g N	Applied 1 g N	Drought duration	Sampled and rewatering Applied 3 g N

### Yield and yield components

At the mature stage, seven rice plants with consistent growth were selected from each treatment group, and their panicles, leaves and stems were all separated. The yield per plant and yield components (number of effective panicles per plant, total grains per panicle, seed setting rate, panicle length, 1000-grain weight) were investigated after air-drying in the sun.

### Dry matter accumulation, distribution and translocation

In each treatment, five rice plants with the same growth were selected at the heading stage and harvest stage, and their leaves, panicles, and stems were all separated and packed for later grinding. Then put them in an oven at 80 °C and dried to constant weight. At the mature stage, the panicles were naturally dried in the sun to constant weight and weighed uniformly. The export percentage of stem and sheath matter (EPMSS) and transformed percentage of stem and sheath matter (TPMSS) were calculated based on the literature [59].

### Calculation of nitrogen accumulation

The nitrogen content was determined according to the micro-Kjeldahl method [60]. The organs of the rice (whole plant stem, leaf, and panicle) were crushed by a high-speed pulverizer (Zhejiang Ronghao Industry and Trade Co. Ltd., China), and a 0.5 g powdered sample was heated and digested with sulfuric acid and a catalyst and analyzed with a FOSS nitrogen determinator (FOSS Kjeltec 8400, Danmephus Analytical Instrument Co., Ltd.).

### SPAD value

The SPAD value of the top second leaf was measured by a hand-held SPAD-502 chlorophyll meter (Chlorophyll Meter SPAD-502 Plus, Zhejiang Tuopu Science and Technology Co. Ltd., China). After the drought stress, five rice plants with consistent growth were selected and labeled for each treatment. The average value of the leaf top, leaf middle and leaf base was taken as the SPAD value of the leaf.

### Net photosynthetic rate

Determination of the net photosynthetic rate after the end of drought. The net photosynthetic rate of leaves was determined using a CI-340 photosynthesizer (CID Bio-Science, USA) from 9:00 to 11:00 a.m. on a clear day. In each treatment, the top second leaves of 5 plants with good and consistent growth were selected to mark and measure.

### Osmotic regulatory substance and enzymatic activity measurements

The leaf samples were collected on the same day after the end of drought treatment. The determination principles of each osmotic regulatory substance were as follows: the anthrone colorimetric method was used for the determination of soluble sugars [61], the soluble protein content [62] was determined by the BCA method, the malondialdehyde (MDA) content [63] was determined by the thiobarbituric acid method, and the extraction of free proline [64] was done according to the sulfosalicylic acid method (The kits used in the experiment were provided by Suzhou Keming Biotechnology Co., Ltd).

The step for determining enzyme activity was to 0.1 g leaf samples from the top second leaf of the main stem of three rice plants were taken from each treatment group. Then, the leaf samples were directly frozen in liquid N and placed in a − 80 °C freezer for storage. The activities of nitrate reductase (NR) [65], glutamine synthetase (GS) [66] and glutamic dehydrogenase (GDH) [62] were determined with kits (COMIN, Suzhou, China).

### Metabolite extraction and data analysis

The leaves of rice with different nitrogen fertilizer applications were sampled at the first day after the end of the drought. Eight biological replicates were taken for each treatment group. 80 mg of each sample of NAD and NBD was accurately weighed, added to the internal standard and ground; 200 μl of the supernatant was extracted after centrifugation for 15 min and stored at low temperature until liquid chromatograph-mass spectrometry (LC-MS) determination. Throughout the LC-MS analysis, QCs were injected into every 8 samples to evaluate the repeat ability of the analysis. The conversion software program MS converter was used to convert the raw data to common data format files (mzML), and the peak extraction of metabolic data was performed by XCMS software (version: 1.50.1). Then, after visual examination of the base peak chromatogram of all leaf samples, the variables with relative standard deviations (RSDs) of more than 30% in the QC samples were further removed. In total, 3882 positive ions and 3199 negative ions were obtained after ion filtering, the screening rates were 89.91 and 94.95%, respectively. The feature yield was more than 75% proved that the metabolomics method was reliable.

### Protein extraction and data analysis

NBD and NAD were performed in three biological replicates (each repeated selection was of the same barrel of plants). For the extraction of proteins, the sample was ground into powder with liquid nitrogen, and then the proteins were digested by enzyme hydrolysis according to the extraction process of the protein sample [67]. The protein concentration measurement was carried out by using the BCA kit after extraction. At the same time, the protein band of each protein sample, visualized via SDS-PAGE electrophoresis, was clear and uniform, the protein was not degraded, and each lane had good parallelism (Fig. S4). The samples were then analyzed by post-reverse chromatography mass spectrometry using Agilent 1100 HPLC. Proteome Discoverer™ 2.2 (Thermo, USA) was used to analyze the data obtained from the experiment, and the database used was the Uniprot GOA rice database (www.http://www.ebi.ac.uk/GOA/).

### Statistical analysis

SPSS 19.0 (v20.0, SPSS Inc., Chicago, USA) and Origin 9.0 for significant difference analysis and and plotting; *p* < 0.05 represented a significant variance test result.

## Supplementary Information

**Additional file 1: Figure S1.** DEPs protein interaction analysis of ‘Wufengyou 286’ under heavy nitrogen application before and after drought.

**Additional file 2: Figure S2.** Multivariate statistical analysis score plots and response ranking test plots of ‘Wufengyou 286’ under heavy nitrogen application before and after drought. (A) Principal component analysis; (B) PLS-DA; (C) OPLS-DA; (D) Response ranking check diagram for OPLS-DA models.

**Additional file 3: Figure. S3.** Hierarchical clustering of metabolites changes in ‘Wufengyou 286’ under heavy nitrogen application before and after drought. The columns correspond to repeats of the NAD and NBD groups and the rows correspond to differential metabolites. Red and green represent high and low expression of DMs for NBD and NAD, respectively. For more information on metabolites, please refer to Supplementary Dataset S2.

**Additional file 4: Figure S4.** Detection of total protein in ‘Wufengyou 286’ under heavy nitrogen application before and after drought by SDS-PAGE electrophoresis. NBD-1, NBD-2 and NBD-3; NAD-1, NAD-2 and NAD-3 represent three repeats of different treatments.

**Additional file 5: Dataset S1.** Information on DEPs identified of ‘Wufengyou 286’ under heavy nitrogen application before and after drought.

**Additional file 6: Dataset S2.** Information on DMs identified of ‘Wufengyou 286’ under heavy nitrogen application before and after drought.

## Data Availability

All data generated or analysed during this study are included in this published article and its supplementary information files.

## Abbreviations

NBD: Heavy nitrogen application before drought
NAD: Heavy nitrogen application after drought
SPAD: Soil and plant analyzer development
EPMSS: Export percentage of stem and sheath matter
TPMSS: Transformed percentage of stem and sheath matter
MDA: Malondialdehyde
SOD: Superoxide dismutase
NR: Nitrate reductase
GS: Glutamine synthetase
GDH: Glutamic dehydrogenase
PCA: Principal component analysis
(O)PLS-DA: (orthogonal) partial least-squares-discriminant analysis
DMs: Differential metabolites
DEPs: Differentially expressed proteins
KEGG: Kyoto encyclopedia of genes and genomes
