# Supplementary material for: Combined proteomics, metabolomics and physiological analyses of rice growth and grain yield with heavy nitrogen application before and after drought
Source: BMC Plant Biol. 2020 Dec 10;20:556. doi: 10.1186/s12870-020-02772-y (PMC7731554; doi:10.1186/s12870-020-02772-y)

**Fig. S1** DEPs protein interaction analysis of ‘Wufengyou 286’ under heavy nitrogen application before and after drought.


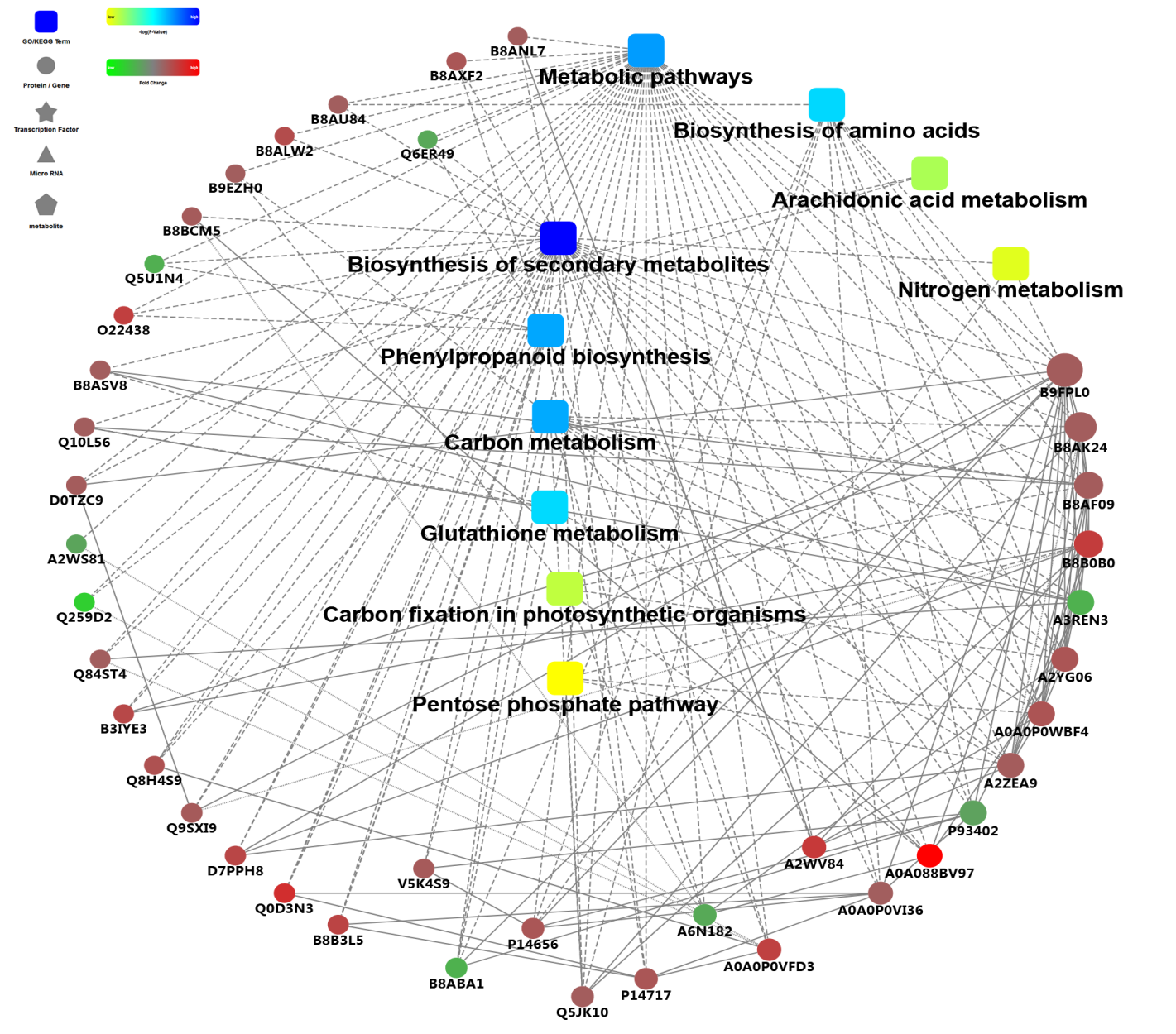

Supplement: Supplementary file 1 — Additional file 1: Figure S1. DEPs protein interaction analysis of ‘Wufengyou 286’ under heavy nitrogen application before and after drought. [file 12870_2020_2772_MOESM1_ESM.docx]
