# Supplementary material for: Combined proteomics, metabolomics and physiological analyses of rice growth and grain yield with heavy nitrogen application before and after drought
Source: BMC Plant Biol. 2020 Dec 10;20:556. doi: 10.1186/s12870-020-02772-y (PMC7731554; doi:10.1186/s12870-020-02772-y)

**Fig. S2** Multivariate statistical analysis score plots and response ranking test plots of ‘Wufengyou 286’ under heavy nitrogen application before and after drought. (A) Principal component analysis; (B) PLS-DA; (C) OPLS-DA; (D) Response ranking check diagram for OPLS-DA models


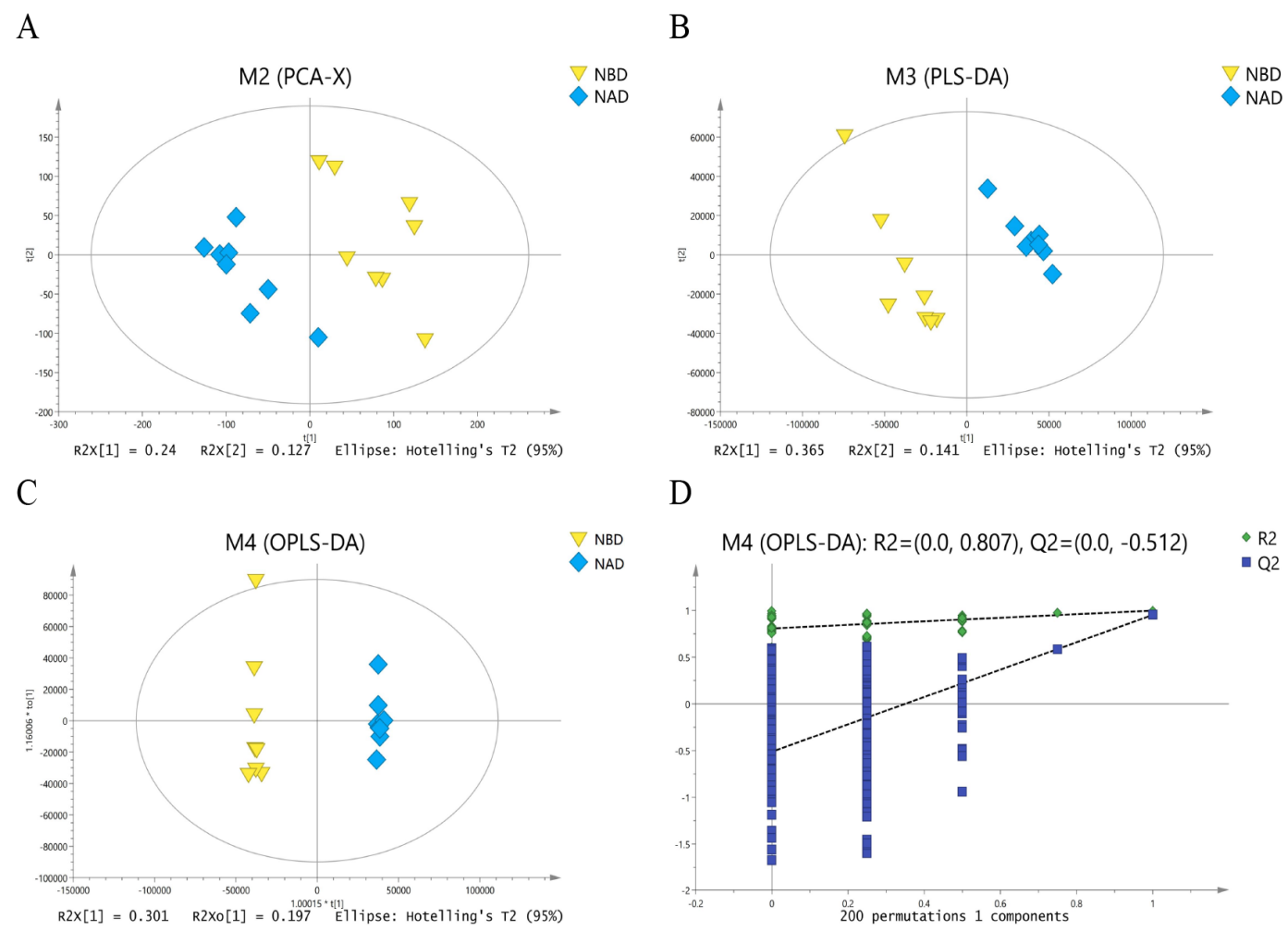

Supplement: Supplementary file 2 — Additional file 2: Figure S2. Multivariate statistical analysis score plots and response ranking test plots of ‘Wufengyou 286’ under heavy nitrogen application before and after drought. (A) Principal component analysis; (B) PLS-DA; (C) OPLS-DA; (D) Response ranking check diagram for OPLS-DA models. [file 12870_2020_2772_MOESM2_ESM.docx]
