# Supplementary material for: Combined proteomics, metabolomics and physiological analyses of rice growth and grain yield with heavy nitrogen application before and after drought
Source: BMC Plant Biol. 2020 Dec 10;20:556. doi: 10.1186/s12870-020-02772-y (PMC7731554; doi:10.1186/s12870-020-02772-y)

**Fig. S3** Hierarchical clustering of metabolites changes in ‘Wufengyou 286’ under heavy nitrogen application before and after drought. The columns correspond to repeats of the NAD and NBD groups and the rows correspond to differential metabolites. Red and green represent high and low expression of DMs for NBD and NAD, respectively. For more information on metabolites, please refer to Supplementary Dataset S2


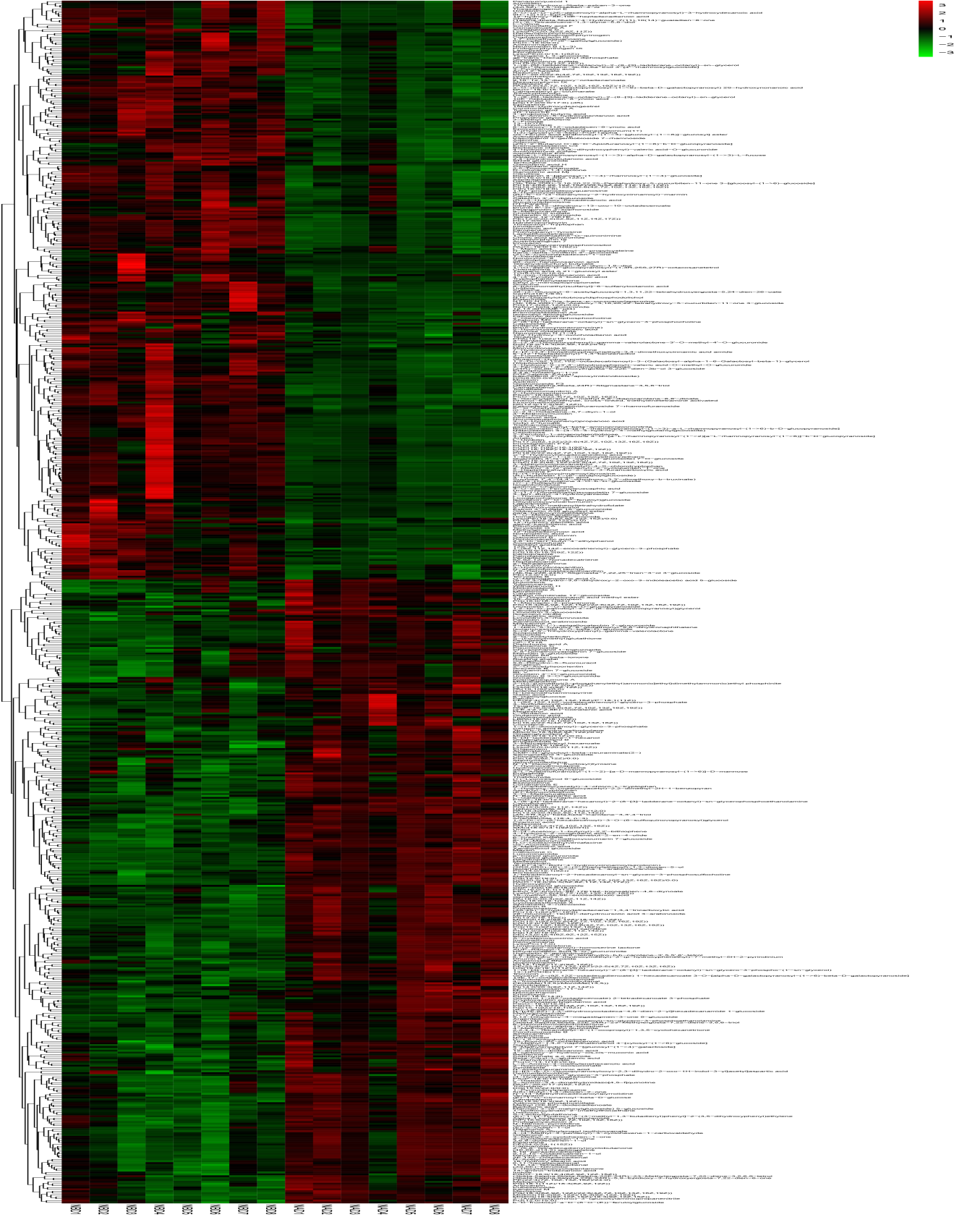

Supplement: Supplementary file 3 — Additional file 3: Figure. S3. Hierarchical clustering of metabolites changes in ‘Wufengyou 286’ under heavy nitrogen application before and after drought. The columns correspond to repeats of the NAD and NBD groups and the rows correspond to differential metabolites. Red and green represent high and low expression of DMs for NBD and NAD, respectively. For more information on metabolites, please refer to Supplementary Dataset S2. [file 12870_2020_2772_MOESM3_ESM.docx]
