# Supplementary material for: Combined proteomics, metabolomics and physiological analyses of rice growth and grain yield with heavy nitrogen application before and after drought
Source: BMC Plant Biol. 2020 Dec 10;20:556. doi: 10.1186/s12870-020-02772-y (PMC7731554; doi:10.1186/s12870-020-02772-y)

**Fig. S4.** Detection of total protein in ‘Wufengyou 286’ under heavy nitrogen application before and after drought by SDS-PAGE electrophoresis. NBD-1, NBD-2 and NBD-3; NAD-1, NAD-2 and NAD-3 represent three repeats of different treatments.


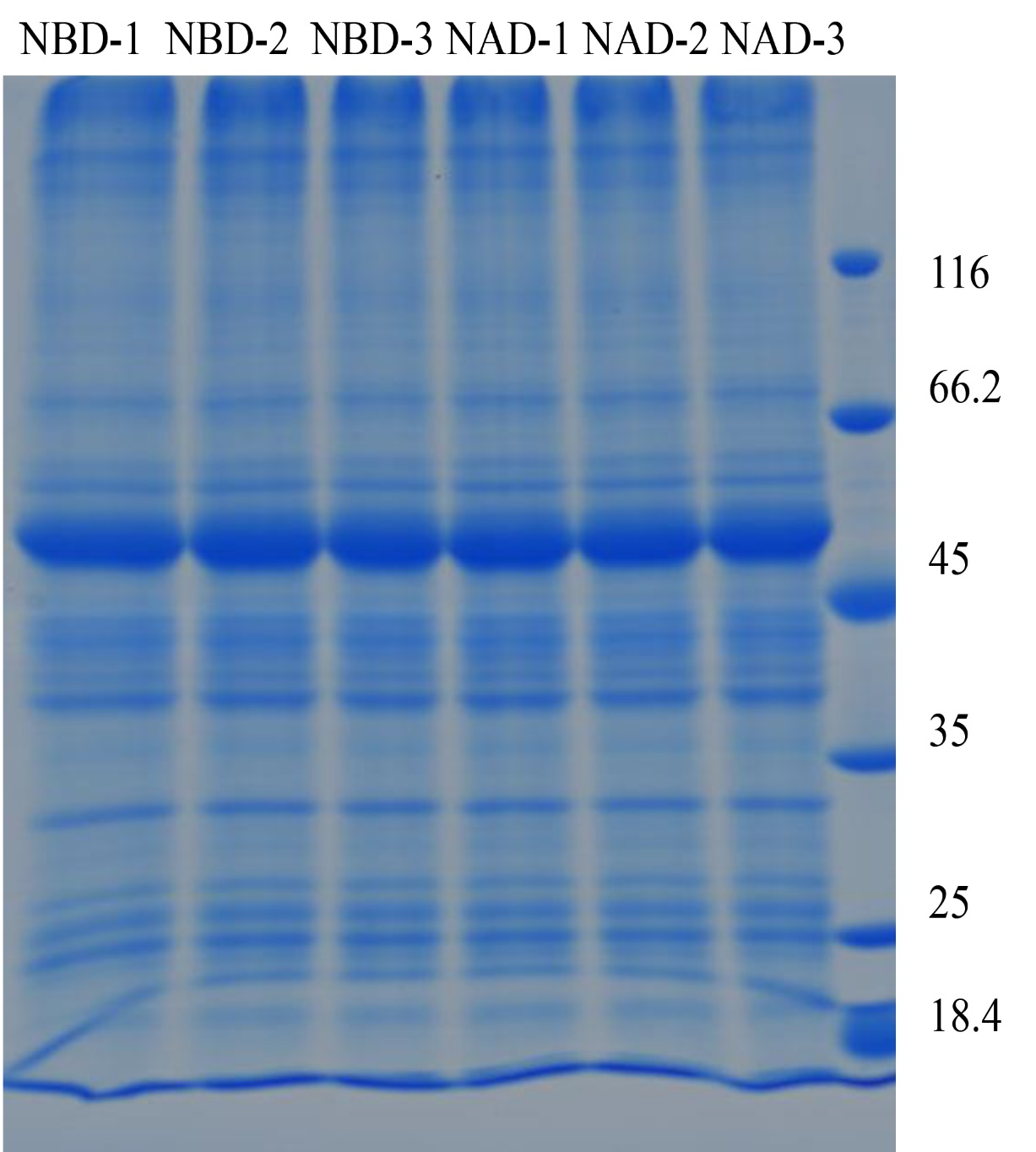

Supplement: Supplementary file 4 — Additional file 4: Figure S4. Detection of total protein in ‘Wufengyou 286’ under heavy nitrogen application before and after drought by SDS-PAGE electrophoresis. NBD-1, NBD-2 and NBD-3; NAD-1, NAD-2 and NAD-3 represent three repeats of different treatments. [file 12870_2020_2772_MOESM4_ESM.docx]
